# Assessment of Safe Drinking Water Handling Practices in Households of Northern India: A Cross-Sectional Study

**DOI:** 10.7759/cureus.55888

**Published:** 2024-03-10

**Authors:** Deepanshi Saxena, Lokesh Raheja, Raja Rao Tamma, Pankaj K Jain, Nilima Takhelchangbam

**Affiliations:** 1 Epidemiology and Public Health, Sarojini Naidu Medical College, Agra, IND; 2 Community Medicine, Amar Shaheed Jodha Singh Attaiya Thakur Dariyao Singh Medical College, Fatehpur, IND; 3 Community Medicine, Umanath Singh Autonomous State Medical College, Jaunpur, IND; 4 Community Medicine, Uttar Pradesh University of Medical Sciences, Etawah, IND; 5 Community Medicine, Rani Durgawati Medical College, Banda, IND

**Keywords:** community health, public and environmental health, handwashing practice, environment health, wash, preventive health, developing countries, sanitation

## Abstract

Background

Waterborne diseases are the most common form of infectious disease, spreading from contaminated water, especially in a developed country. These diseases are a major concern for the environment and public health. The living conditions in developing countries like India affect the water-handling practices, which make the population vulnerable to waterborne diseases. The inability to access safe drinking water also adds to this. Water safety for a community relies on water collection, treatment, storage, and handling in the household setting. Therefore, the burden of waterborne disease can be reduced by treating point-of-use drinking water, including improving handling and transport.

Objectives

The aim was to assess the safe drinking water handling practices in households. The objectives were to assess the safe drinking water-handling practices, namely, treatment, storage, lid status of the storage vessel, and water drawing technique, and to estimate the sources of safe drinking water.

Methods

This cross-sectional study was conducted in the Etawah district on a total of 312 eldest female family members actively working in the kitchen. Descriptive analysis and Chi-Square test were applied to the collected data and a p-value <0.05 at 95% confidence interval (CI) was taken as statistically significant.

Results

Overall, 135 (85.9%) households in urban areas relied on public supply. However, in rural areas mostly 130 (83%) households depended on private supply. In water-handling practices, 276 (88.4%) used some method to purify drinking water, a total of 209 (67%) households kept the lid of the storage container covered, and 249 (79.8%) households drew water either by pouring or scooping with a long handle.

Conclusion

The study concluded that both private and public sources were used for drinking water. Regarding water-handling practices, most households drank purified water, kept their containers covered, and drew water either by scooping or pouring from storage containers. Those who drank purified water mostly belonged to nuclear families and had private sources of drinking water.

## Introduction

Sanitation and safe drinking water are human rights and essential components for economic development and social welfare. Contaminated water spreads potential waterborne diseases, directly or indirectly. Fecal-oral transmission of pathogens causes diarrheal disease, the leading cause of childhood mortality. The link between early pathogen exposure, waterborne diseases, and high rates of stunting, commonly known as environmental enteropathy, is well understood [1, 2]. Water, sanitation, and hygiene (WASH) issues and related initiatives affect children's growth and development [3]. These causes represent 60% of all deaths due to diarrhea globally, including nearly 300,000 children under the age of five, representing 5.3% of all deaths in this age group. It can be reduced by the treatment of point-of-use drinking water, including improvement in handling and transport, and will aid in achieving Sustainable Development Goal 6 [4-6].

Water-handling practices involve ensuring that water remains uncontaminated while passing from the water source to the level of consumption. These crucial preventive stages in water-handling practices involve collecting water from the source, purification or treatment, storage, the storage vessel and its lid status, water-drawing technique, and the cleanliness of the vessel.

During handling, water may get contaminated. Therefore, to ensure it is free from prior chemical and microbiological contamination, and to prevent waterborne diseases, it is a must to understand water-handling practices. Households and communities can improve water quality and maintain quality through good water-management practices. It includes treating water before drinking and improving handling during transportation from the source to the home and while using it domestically. This will also decrease the burden of waterborne disease. Still, people are reluctant to adopt new behaviors that may improve health outcomes and maintain water quality [7].

Hand washing with soap after stool contact is a key barrier to prevent the fecal-oral spread of diarrhea because it prevents germs from entering the household environment and the body. This helps in maintaining appropriate water-handling practices and management [8].

Despite many studies globally, there is a paucity of studies in this location; therefore, the authors conducted this study to assess the safe drinking water handling practices in households in Northern India. The objectives were 1) to assess safe drinking water-handling practices, viz., treatment, storage, lid status of the storage vessel, and water drawing technique, and 2) to estimate the sources of safe drinking water.

## Materials and methods

Study design and study area

This was a community-based cross-sectional study conducted from January 2020 to December 2021 and was part of a larger study. The ethical clearance was taken from the Institutional Ethical Committee (ID-84/2019-20). The urban and rural field practice areas under the Department of Community Medicine, Etawah district, Uttar Pradesh, were selected purposively.

Sample size

Based on data from the National Health Family Survey 4 (NFHS), it was found that 96.4% of households had access to improved drinking water sources [9]. Using this data, we obtained a sample size of 148 households with 3% absolute precision at a 95% Confidence Interval (CI). Accounting for a 5% loss to follow-up (chosen over 10% due to financial constraints), an additional 5% of 148 was added, resulting in a total of 156. This sample size was rounded off. Consequently, 156 samples were selected from both urban and rural areas for comparative analysis, totaling 312 samples.

**Figure 1:**
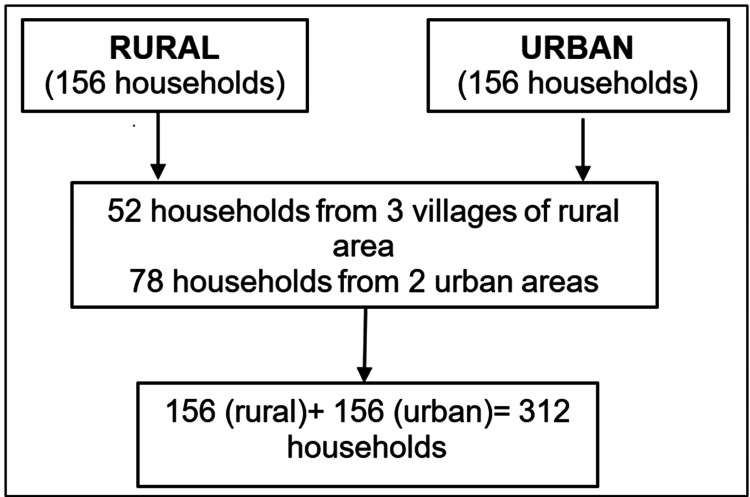
Flowchart of the methodology of data collection

The current updated list of households was obtained from the Community Medicine Department. For selecting the household, a systematic random sampling method was applied until the sample size was achieved and the first house was selected by obtaining a random number using a currency note. From each of the three villages, 52 households were selected for the survey to achieve a total sample of 156 households. Similarly, from each of the two urban areas, 78 households were selected (Figure 1).

Study participants and eligibility criteria

The eldest female of the family was included, actively doing the kitchen chores and the residents of the area. The reason to include females was their major responsibility for these tasks in our country. In the absence of females, the widowers or divorced males or male partners living far from their female partners were included in the study. If the respondents were not available, one revisit was arranged later. If on that day they were unavailable then they were excluded from the study.

Pilot testing of the questionnaire

The data were collected by using a predesigned, pretested, and semi-structured questionnaire. The content of the questionnaire was validated by one of the faculty of the institute. The questionnaire was translated into the local dialect i.e., in Hindi to ensure the equivalent nature of both the questionnaires in Hindi and English, forward translation and back translation methods were applied. To ensure the reliability of this tool the authors conducted a reliability test using Cronbach’s alpha (0.713) on the data of the pilot study for internal consistency.

Data Collection Tool

The questionnaire attached in the Appendices consisted of three sections: first and second - socio-demographic profile. It included name, age, religion, caste, type of family, number of family members, socio-economic status of the family, education, income, marital status, occupation of the study participants, and whether livestock was present and the distance from dwelling houses (ideally, cattle should be kept 25 feet away from the households).

The third section contained questions regarding drinking water sources and management. The question comprises the water source, method of water treatment, storage, type of storing containers, lid status of storage containers, drawing technique of water, frequency of washing containers used for storing drinking water if washing by only water or using soap or detergent and the livestock near the source of water (Table 1).

**Table 1:** Variables and responses

Variables	Responses
Sources of safe drinking water (Refers to the point from which water is collected and not the origin of the water supplied^9,10^)	Private source: Borehole/Submersible; Public source: supplying in the household; Others: from a neighbor and public source, Packaged drinking bottle
Treatment of water	Any purification method: Boiling, reverse osmosis (RO) treatment, Filter
Storage Lid Status	Any storage container Covered/Uncovered
Drawing technique	Pouring into a cup; Scooped with a long handle; Scooped without handle

Operational definition

Here, the drinking water source refers to the point from which water is collected (for example, the tap or borehole/handpump) and not the origin of the water supplied (for example, surface water or groundwater). Safe refers to drinking water from an improved water source (for example, piped water, boreholes or submersibles, and packaged water) [6, 10].

Data Collection

As the duration of data collection was May 2021 to November 2021 during the COVID-19 period, the further procedure of research was delayed up to an extent. Verbal and written consent was taken after a full explanation of the procedure and adhering to COVID-19-appropriate behaviors. If any information was missing for any particulars, the respective study subject was contacted again on the next visit, thus there were no missing data. The data were entered into a Microsoft Excel sheet (Microsoft Corporation, Redmond, USA). Statistical analysis was done by using SPSS Version 25.0 (IBM Corp., Armonk, USA). The descriptive data were reported as frequency and percentage, and the association between variables was determined by Chi-Square and Fisher exact tests wherever applicable. A p-value of less than 0.05 at a 95% confidence interval (CI) was taken as statistically significant.

## Results

There were 156 participants each from rural and urban areas as shown by the sociodemographic characteristics of the study participants (N=312). Most participants (138, 44.2%) in rural and urban areas were between the ages of 26 and 35 years. Nuclear families were more common in the urban (106, 60.5%) than in the rural areas (69, 39.4%) (Table 2).

**Table 2:** Socio-demographic characteristics of the study participants (N=312) *Modified B. G. Prasad Scale (All India Consumer Price Index up to April 2020).

Characteristics		Rural (n=156) Frequency (%)	Urban (n=156) Frequency (%)	Total (N=312)
Age group (In years)	19-25	21(13.5)	19(12.2)	40
	26-35	69(44.2)	69(44.2)	138
	36-45	42(26.9)	43(27.6)	85
	46-55	13(8.3)	14(9.0)	27
	56 and above	11(7.1)	11(7.1)	22
Religion	Hindu	156 ( 53)	138 ( 46.9 )	294
	Muslim	0 ( 0 )	18 ( 100 )	18
Type of family	Nuclear	69 ( 39.4)	106 ( 60.5 )	175
	Joint	51 ( 60.7 )	33 ( 39.2 )	84
	Three generation family	36 ( 67.9 )	17 ( 32.0 )	53
Occupation	Housemaker	148 ( 49. 9)	151 ( 50.5 )	299
	Others: Unskilled	8 ( 61.5 )	5 ( 38.4 )	13
Marital status	Unmarried	18 ( 90 )	2 ( 10 )	20
	Married	132 ( 48 )	143 ( 52 )	275
	Others: Widow	6 ( 35.2 )	11 ( 64.7 )	17
Educational status	Illiterate	42 ( 26.9)	26 ( 38.2 )	68
	Primary school	23 ( 14.7 )	42 ( 64.6 )	65
	Secondary school	80 ( 51.3 )	87 ( 52.0 )	167
	Graduate	11 ( 7.1 )	1 ( 8.3 )	12
Socio-economic status^*^	Upper class	44 ( 28.2 )	57 ( 36.5 )	101
	Upper middle	53 ( 34 )	36 ( 23,1)	89
	Lower middle	42 ( 26.9 )	29 ( 18.6)	71
	Upper lower-	15 ( 9.6 )	23( 14.7 )	38
	Lower	2 ( 1.3 )	11 ( 7.1 )	13

Overall, 135 (85.9%) households in urban areas relied on public supply. However, in rural areas, 130 (83%) households depended on private supply.

This study showed that more households in rural (153, 98.1%) than in urban (123, 78.9%) areas used a method of purification, among which the candle filter was the most common (225, 81.5%) method. The most common (94, 60%) water storage container was a bucket in rural. However, in urban, the most common (67, 43%) water storage container was a bottle or jug. The stored water was covered in maximum (209, 70%) households and mostly in rural households 128 (82.1%). The frequency of cleaning the water storage containers was found to be daily in almost all (143, 65.3%) households in rural areas, while in urban areas, only 77 (34.6%) households reported the same. The most common (249, 79.8%) method of drawing water from the water storage container was pouring/scooping with a long handle in both areas (Table 3).

**Table 3:** Distribution of drinking water sources and water handling practices among households (N=312) *N/A-Not applicable

Variables	Categories	Rural (n=156) Frequency (%)	Urban (n=156) Frequency (%)	Total (N=312)
Sources of drinking water	Private supply: Borehole/ Submersible	130 (83.3)	21 (13.5)	151
	Public supply: supplying in Household	0 (0)	135(85.9)	135
	Others: from neighbors and public sources, Packaged drinking bottle	26 (16.7)	0 (0)	26
Purification status: Boiling, RO treatment, Filter	Yes	153(98.1)	123(78.9)	276
	No	3 (1.9)	33 (21.1)	36
Method of purification (Total=276)	Boiling	4(17.4)	19(82.6)	23
	Electric filter (Reverse osmosis or ultraviolet (U.V.) filter)	10(35.7)	18(64.3)	28
	Candle filter	139(61.8)	86(38.2)	225
Lid status	Covered	128 (82.1)	81 (51.9)	209
	Uncovered	28 (17.9)	75 (48.1)	103
Drawing technique of water from storage	Pouring/scooping with a long handle	97(62.2)	152(97.4)	249
	Scooping without handle	59(37.8)	4(2.6)	63
Frequency of cleaning	Daily	143 (62.7)	85 (37.2)	228
	Weekly	1 (6.7)	14 (93.3)	15
	Biweekly	2 (4.8)	39 (95.1)	41
	*N/A: Water gets stored in the filter	10 (35.7)	18 (64.3)	28
Cleaning agents	Soap/ Detergent plus water	136 (54.6)	113 (45.3)	249
	Only water	10 (28.6)	25 (71.4)	35
	*N/A: Water gets stored in the filter	10 ( 35.7)	18 (64.3)	28

The drainage system was present near most of the households in rural (91, 58 %) and urban (133, 85%) areas. Household waste or livestock was present near sources of drinking water in rural areas in 87 (56%) households, whereas they were absent in 119 (76%) households in urban areas.

Out of 151 households that had private sources as a source for drinking water, 146 (96.7%) households used some method of purification of drinking water, and the association was found to be statistically significant (p-value <0.05 at CI 95%). Out of 209 households that kept their lids covered, 175 (83.7%) households used some method of purification of drinking water, and the difference was found to be statistically significant (p-value <0.001). From a total of 249 households who drew water either by pouring/scooping with a long handle 216 (86.7%) used some method of purification, and the difference was found to be statistically significant (p-value <0.05). The association between the method of purification of drinking water and the drawing technique of water from storage was found to be statistically significant (p-value =0.05 at CI 95%) (Table 4.)

**Table 4:** Association between drinking water purification method, and other variables (N=312) *Chi-square and Fischer exact test applied

Age groups (In years)	Categories	Purification method		Total (N=312)	p-value^*^
		Yes	No		0.03
	19-25	34 (85.0)	6 (15.0)	40	
	26-35	128(92.8)	10(7.2)	138	
	36-45	68(80.0)	17(20.0)	85	
	46-55	25(92.6)	2(7.4)	27	
	56 and above	21(95.5)	1(4.5)	22	
Type of family	Nuclear	150(86.2)	25(14.3)	174	0.01
	Joint	80(95.2)	4(4.8)	84	
	Three generation family	46(86.8)	7(13.5)	52	
Educational status	Illiterate	62(91.2)	6(8.8)	68	0.08
	Primary school	52(80.0)	13(20.0)	65	
	Secondary school	150(89.8)	17(10.2)	167	
	Graduate	12(100.0)	0	12	
Socioeconomic status	Upper class	91(90.1)	10(9.9)	101	0.2
	Upper middle	80(89.9)	9(10.1)	89	
	Lower middle	62(87.3)	9(12.7)	71	
	Upper lower	34(89.5)	4(10.5)	38	
	Lower	9(69.2)	4(30.8)	13	
Sources of drinking water	Private source: Borehole/ Submersible	146(96.7)	5(3.3)	151	<0.001
	Public source: supplying in Household	104(77.0)	31(23.0)	135	
	Others: from neighbors and public sources, Packaged drinking bottle	26(100.0)	0	26	
Lid Covered	Yes	175(83.7)	34(16.3)	209	<0.001
	No	101(98.1)	2(1.9)	103	
Drawing technique of water from storage	Pouring/scooping with a long handle	216(86.7)	33(13.3)	249	0.05
	Scooping without handle	60(95.2)	3(4.8)	63	

The results revealed that out of 175 households, 99 (56.6%) households that were nuclear families kept the lids of water storage containers covered, and the association was found to be statistically significant (p-value <0.001). Out of a total of 151 households, 128 (84.8%) households taking water from private sources kept the lids of water storage containers covered, and the association was found to be statistically significant (p-value <0.001) (Table 5).

**Table 5:** Association between lid status of water storage container, and other variables of the study (N=312) *Chi-square and Fischer exact test applied

Variables	Categories	Lid covered		Total (N=312)	P-value^*^
Age groups (In years)		Yes	No		
	19-25	30(75.0)	10(25.0)	40	0.2
	26-35	96(69.6)	42(30.4)	138	
	36-45	57(67.1)	28(32.9)	85	
	46-55	14(51.9)	13(48.1)	27	
	56 and above	12(54.5)	10(45.5)	22	
Type of family	Nuclear	99(56.6)	76(43.4)	175	<0.001
	Joint	64(76.2)	20(23.8)	84	
	Three generation family	46(86.8)	7(13.2)	53	
Educational status	Illiterate	48(70.6)	20(29.4)	68	0.1
	Primary school	36(55.4)	29(44.6)	65	
	Secondary school	115(68.9)	52(31.1)	167	
	Graduate	10(83.3)	2(16.7)	12	
Socioeconomic status of the family	Upper class	57(56.4)	44(43.6)	101	0.012
	Upper middle	60(67.4)	29(32.6)	89	
	Lower middle	57(80.3)	14(19.7)	71	
	Upper lower	24(63.2)	14(36.8)	38	
	Lower	11(84.6)	2(15.4)	13	
Source of water	Private source: Borehole/ Submersible	128(84.8)	23(15.2)	151	<0.001
	Public source: supplying in Household	61(45.2)	74(54.8)	135	
	Others: from neighbors and public sources, Packaged drinking bottle	20(76.9)	6(23.1)	26	
Drawing technique of water from storage	Pouring/scooping with a long handle	156(62.7)	93(37.3)	249	<0.001
	Scooping without handle	53(84.1)	10(15.9)	63	

Out of 175 nuclear families, 138 (78.9%) drew water either by pouring or scooping with a long handle, and the association was found to be statistically significant (p-value <0.001). Out of 135 households that took water from public sources, 131 (97%) households drew water either by pouring or scooping with a long handle, and the association was found to be statistically significant (p-value <0.001) (Table 6).

**Table 6:** Association between drawing technique of water from the storage container, and other variables of the study (N=312) *Chi-square and Fischer exact test applied

Variables	Categories	Drawing technique of water from storage container		Total (N=312)	P-value^*^
		Pouring/scooping with a long handle	Scooping without handle		
Age groups (In years)	19-25	33(82.5)	7(17.5)	40	0.7
	26-35	112(81.2)	26(18.8)	138	
	36-45	64(75.3)	21(24.7)	85	
	46-55	21(77.8)	6(22.2)	27	
	56 and above	19(86.4)	3(13.6)	22	
Type of family	Nuclear	138(78.9)	37(21.1)	175	<0.001
	Joint	75(89.3)	9(10.7)	84	
	Three generation family	36(67.9)	17(32.1)	53	
Educational status	Illiterate	53(77.9)	15(22.1)	68	0.5
	Primary school	54(83.1)	11(16.9)	65	
	Secondary school	134(80.2)	33(19.8)	167	
	Graduate	8(66.7)	4(33.3)	12	
Socioeconomic status	Upper class	87(86.1)	14(13.9)	101	0.06
	Upper middle	70(78.7)	19(21.3)	89	
	Lower middle	49(69.0)	22(31.0)	71	
	Upper lower	31(81.6)	7(18.4)	38	
	Lower	12(92.3)	1(7.7)	13	
Source	Private source: Borehole/ Submersible	100(66.2)	51(33.8)	151	<0.001
	Public source: supplying in household	131(97.0)	4 (3)	135	
	Others: from neighbor and public source, Packaged drinking bottle	18(69.2)	8(30.8)	26	

To sum up, this study demonstrated only 135 (43.2%) households had public supply as a source of drinking water in both areas. However, almost all households had access to safe drinking water in both areas. Overall, 276 (88.5%) households used some method of purification of drinking water. A total of 249 (79.8%) households drew water either by pouring or scooping with a long handle.

## Discussion

Considering the importance of safe drinking water management practices, this study was conducted. In this study, 156 households from both rural and urban areas participated. In rural areas, the majority, 130 (83%) of households took drinking water from private supplies, whereas, in urban areas, the public supply for drinking water was found to be the most common (135, 85.9%). More focus should be given to the water supply in the rural areas. The present study revealed that the proportion of households having access to safe drinking water in field practice areas was almost 100%. The NFHS-5 (2019-21) published a report revealing that 99.5% of households in the Etawah district of Uttar Pradesh had access to safe drinking water, which is similar to this study [9].

This study revealed that 88.4% (276) of households used some method of drinking water purification. In the study conducted by Joshi A. et al. in the urban slums of New Delhi in the year 2014, Kuberan A. et al. [11] in Thandalam village, Chennai, and by Pachori R. et al. [12] in Tamil Nadu, the proportion of accessibility to safe drinking water was 100%, 99%, and 85.3%, respectively. Similarly, in the study conducted by Bhar et al. [1] in the slum households of Siliguri Municipal Corporation and by Kong et al. [14] in urban and rural localities of Malaysia, a developing country like India, the proportion of accessibility to safe drinking water was 92.1% and 96.2%, respectively. This may be due to the proper government pipeline supply of drinking water. Hence, the results of the various studies showed strong political commitment and determined implementation of the program by the government [1, 11-14].

The results of this study showed that in water handling practices in the district of Uttar Pradesh, northern India. The most common water storage containers were buckets or jugs (wide-mouthed) and bottles (narrow-mouthed). Water was found covered in most households (209, 67%). In the other water handling practices; lid coverage was significantly (p<0.005) associated with the type of family, socioeconomic status, source of drinking water, and drawing technique of water from storage. Maybe nuclear families give more focus to the health of their children; therefore, following appropriate water handling practices was observed. Joshi A. et al. [13] also showed slightly different preferences of the household for containing the drinking water, i.e., narrow-mouthed container (63%), and keeping it covered. Kuberan A. et al. [11] revealed that most (75%) households stored water in a wide-mouthed covered container. Ssemugabo et al. [15] conducted the study in slum communities, in Kampala, Uganda, and showed that most (97%, 383) of the people were using a narrow-mouthed container to store water. Pachori R et al. [12] also showed in their study similar results (256, 85.3%). The choices are individual for any household and depend on the ease of access [11-13, 15].

Surprisingly, in the present study, the majority (222, 71.1%) cleaned the water storage container daily with soap and water. The study by Reshma et al. [16] reveals almost the same findings. In this study, 83.7% of people practiced washing water storage containers. Joshi et al. [13] showed a similar finding - the majority cleaned it daily. Kuberan et al. [11] revealed similar findings (70%). Their awareness regarding clean or safe water had an impact on their water-handling practices. In this study, 249 out of 312 (79.8%) participants drew water using a long-handled cup from the water storage container. The drawing technique of water from the storage container was associated with the source and type of family. The results by Reshma et al., [16] showed different results - 33.7%. This may be due to the smaller number of family members in nuclear families, allowing the homemaker to follow appropriate drawing techniques and focus more on the cleanliness of drinking water. Additionally, another reason can be the awareness regarding the cleanliness of water storage containers, which was further linked with safe water.

Around 36 (11.5%) households were not using any method for the purification of drinking water in this study. It may be due to the taste preference for water or the preconception that the water was clean. In a study by Ghazanfar et al. [17], the same results were obtained (77%). The practice of purifying water was found to be associated with the age group, type of family, the source of drinking water, and the water-handling practice, such as lid coverage and drawing technique of water from storage. The drawing technique of drinking water from a storage container was found to be significantly associated with the type of family, educational status, the source of drinking water, the lid status of the storage container. This suggests that with higher educational levels, the people were more of the drawing technique of water from the drinking water storage container.

## Conclusions

In this study, 156 households from both rural and urban areas participated. The study concluded that in rural areas, the majority of households took drinking water from a private supply. In the urban area, the households relied on the public supply of drinking water. This study also highlighted that the proportion of households having access to safe drinking water in both areas was approximately 100%, according to the operational definitions. The majority of households in both rural and urban areas drank purified water. The candle filter was the most common choice for all the households. 

Regarding the other water-handling practices, the study participants kept the lid of the drinking water storage container covered. The households were aware enough to wash their drinking water storage containers daily.

The long-handled cup was the most commonly used method for drawing the water from the storage container. Those who drank purified water mostly belonged to nuclear families had private sources of drinking water, kept their storage containers covered, and drew drinking water either by pouring or scooping with a long handle from the storage container. As the socioeconomic status improved, the storage containers were found to be covered, and this difference was significantly associated.

With an increase in educational status, more households drew drinking water either by pouring or scooping with a long handle, and this difference was found to be statistically significant. The study illustrates that the population was following water-handling practices. This shows that people have started acting consciously towards their health. A little effort towards maintaining appropriate safe water-handling practices can prevent various waterborne diseases. There is still a demand for a robust awareness campaign regarding water-handling practices and cost-effective purification techniques to achieve the Sustainable Development Goals of health and well-being and clean water and sanitation for all. Access to safe drinking water and proper water-handling practices are important for public health as their role is crucial in improving the countries’ economic growth, especially the developing ones, as well as contributing greatly to reducing waterborne disease burden, thus reducing poverty.

Limitations

The results of this study should be interpreted considering the study’s limitations. This study was carried out in field practice areas where major interventions are undertaken periodically by the Department of Community Medicine; for example, awareness campaigns regarding waterborne diseases, hygiene, etc. These campaigns may have affected the practices of drinking water storage and handling. Therefore, the findings of the study can’t be generalized. The effect of modifiers or confounders wasn’t taken into account. Further studies can be planned to determine the health outcomes in terms of diarrhoeal load and other health-related issues related to drinking water handling practices can be assessed.

## Appendices

**Title:** “Assessment of Drinking Water Treatment, Storage, and Handling Practices in Etawah District: A Cross-Sectional Study”

Form Serial No. ______

Date:___________

Area:___________

**Table 7:** Socio-demographic details of the participants

S.N.	SECTION A: Particulars
A1	Name of Participant (Confidential)
A2	Husband’s Name (Confidential)
A3	Age of Participant (years)
A4	Name of head of family (Confidential)
A5	Name of area
A6	Contact/Phone no.
	SECTION B: Details
B1	Religion 1. Hindu 2. Muslim 3. Sikh 4. Christian 5. Others (specify)
B2	Caste 1. General 2.OBC 3.SC 4.ST 5. Others (specify)
B3	Type of family 1. Nuclear 2.Joint 3.Three generation family
B4	Educational status 1. Illiterate 2. Primary school 3. Middle school 4. High school 5. Intermediate 6. Graduate7.Postgraduate
B5	Occupation 1. Housewife 2. Unskilled 3. Semiskilled 4. Skilled 5. Semiprofessional/ Professional 6. Retired
B6	Number of family members
B7	Total monthly income of head / own (in Rupees)
B8	Per capita income (in Rupees)
B9	Socioeconomic status of the family according to Modified B G Prasad Scale (AICPI up to APRIL 2020) 1. Upper class- 7510 and above 2. Upper middle-3755-7509 3. Lower middle- 2253-3754 4. Upper lower- 1126-2252 5. Lower < 1125
B10	Type of diet 1. Vegetarian 2. Non-vegetarian
B11	Marital status 1. Unmarried 2. Married 3. Others (specify)
B13	House Status 1. Owner 2.Rented
B14	Livestock 1. Present 2. Absent

**Table 8:** Drinking water sources and water handling practices (interview and observation of practices)

S.N.	SECTION- C: Questions
C1	What is the primary source of drinking water? 1. Private source 2. Public source: supplying in household 3. others (from neighbor and public sources, Packaged drinking bottle)
C2	Drainage system near primary source 1. Present 2. Absent
C3	Household waste or livestock near the primary source (observe) 1. Present 2. Absent
C4	What method do you use for the Purification of drinking water? 1. Boiling 2. Filter/RO 3. Filter with candle 4.No method 5.Others(specify)
C5	Do you store drinking water? (If the answer is yes, then move to C6) 1. Yes, purified water is stored 2. Yes, but water is not purified 3. No, take directly from the source for drinking every time
C6	Where do you store it? 1. Reusable plastic jar 2. Bucket 3. Bottle 4. Jug 5. Stored in the filter itself 6. Water tank 7.Others(specify)
C7	Observe the Lid status of the container used for storing drinking water 1. Covered 2. Partially covered 3. Uncovered
C8	How do you collect stored drinking water to drink? 1. Pouring into cup 2. Scooping into cup a. Scooped with long handle b. Scooped without handle
C9	What is the frequency of cleaning containers used for storing drinking water? 1. Daily 2. Weekly 3. Biweekly 4. Monthly 5. >Monthly to yearly
C10	What do you use to clean the container? 1. Soap / Detergent and water. 2. Only water
